# A Conceptual Framework for Healthy Eating Behavior in Ecuadorian Adolescents: A Qualitative Study

**DOI:** 10.1371/journal.pone.0087183

**Published:** 2014-01-29

**Authors:** Roosmarijn Verstraeten, Kathleen Van Royen, Angélica Ochoa-Avilés, Daniela Penafiel, Michelle Holdsworth, Silvana Donoso, Lea Maes, Patrick Kolsteren

**Affiliations:** 1 Department of Public Health, Institute of Tropical Medicine, Antwerp, Belgium; 2 Department of Food Safety and Food Quality, Ghent University, Ghent, Belgium; 3 Food, Nutrition and Health program, Universidad de Cuenca, Cuenca, Ecuador; 4 Rural Research Centre, Escuela Superior Politécnica del Litoral, Guayaquil, Ecuador; 5 Public Health Section, School of Health and Related Research (ScHARR) - The University of Sheffield, Sheffield, United Kingdom; 6 Department of Public Health, Ghent University, Ghent, Belgium; University of Western Brittany, France

## Abstract

**Objective:**

The objective of this study was to identify factors influencing eating behavior of Ecuadorian adolescents - from the perspective of parents, school staff and adolescents - to develop a conceptual framework for adolescents' eating behavior.

**Study design:**

Twenty focus groups (*N = *144 participants) were conducted separately with adolescents aged 11–15 y (n (focus groups)  = 12, *N* (participants)  = 80), parents (n = 4, *N* = 32) and school staff (n = 4, *N* = 32) in rural and urban Ecuador. A semi-structured questioning route was developed based on the ‘Attitude, Social influences and Self-efficacy’ model and the socio-ecological model to assess the relevance of behavioral and environmental factors in low- and middle-income countries. Two researchers independently analyzed verbatim transcripts for emerging themes, using deductive thematic content analysis. Data were analyzed using NVivo 8.

**Results:**

All groups recognized the importance of eating healthily and key individual factors in Ecuadorian adolescents' food choices were: financial autonomy, food safety perceptions, lack of self-control, habit strength, taste preferences and perceived peer norms. Environmental factors included the poor nutritional quality of food and its easy access at school. In their home and family environment, time and convenience completed the picture as barriers to eating healthily. Participants acknowledged the impact of the changing socio-cultural environment on adolescents' eating patterns. Availability of healthy food at home and financial constraints differed between settings and socio-economic groups.

**Conclusion:**

Our findings endorse the importance of investigating behavioral and environmental factors that influence and mediate healthy dietary behavior prior to intervention development. Several culture-specific factors emerged that were incorporated into a conceptual framework for developing health promotion interventions in Ecuador.

## Introduction

Obesity and chronic diseases are no longer exclusive to affluent societies, but are now the leading cause of morbidity and mortality in low- and middle-income countries (LMICs) [1]. A staggering rise in unhealthy body weight has been observed in children in LMICs across all levels of socio-economic status [2], [3]. This rise is associated with rapid economic and societal changes [4], [5] and has led to obesity prevalence estimates in some LMICs as high as those in high-income countries (HICs) [6]. Prevention is crucial, as childhood obesity is associated with several chronic conditions in adulthood [7]–[9] and premature mortality [10] thereby aggravating the burden on health systems and hindering economic development.

School-based interventions targeting physical inactivity and unhealthy eating are an important strategy in obesity prevention [11]. However, evidence is needed from LMICs of the pathways through which school-based interventions mediate physical activity and dietary behavior [12]. To increase our understanding, intervention studies incorporating theoretical models to address population-specific behavioral and environmental influences on dietary and physical activity behavior are required [13]. Current models may not be transferable to LMICs because culture-specific influences on these behaviors, such as social values/norms and physical environment may be different from HICs.

To develop a conceptual framework for health promotion interventions in Ecuadorian adolescents that accounts for its cultural context, we solicited opinions of adolescents, parents and school staff, using focus groups to explore factors of adolescents' eating behavior.

## Methods

### Ethics statement

Focus groups were conducted between April - September 2008. They were framed within a larger research study and the study protocol was approved by both the Ethics Committees of Quito and the Ghent University Hospital (CBM/cobi-001; B67020084010; 2008/462). The different audiences included in these focus groups were asked for their consent. Adolescents who returned signed parental consent forms and gave written assent to participate were included in the study; parents and school staff needed to provide written consent. The ‘Consolidated criteria for reporting qualitative research checklist’ was used to report the results [14].

### Theoretical framework

Dietary behavior in young people is determined by the complex interplay of factors at both individual and environmental level. To better understand these factors in Ecuadorian adolescents, we used a theoretical framework to conceptualize and analyze the findings of focus group discussions. To ensure the cultural appropriateness of this framework, the cognitive variables from the ‘Attitude, Social influences and Self-efficacy’ ASE-model [15] were nestled within the socio-cultural and physical context of adolescents’ environment, as elaborated by the socio-ecological model [16]. The ASE-model poses that dietary behavior is a function of the intention to perform the behavior that, in turn, can be explained through 3 cognitive factors: attitudes, social influence (including subjective norms, modeling and support) and self-efficacy. Additionally, barriers and lack of skills might limit the possibility to put the intention into practice [17]. As adolescents' dietary behavior is strongly influenced by their environments [18], we complemented our framework with a socio-ecological perspective. In this model, dietary behavior is viewed as the interaction between, and interdependence of, factors within and across multiple levels of influence. In other words, it highlights people's interactions with their physical and socio-cultural environments [16]. Both models have been used extensively to study dietary behaviors in young people [19]–[21].

### Focus groups

The protocol incorporated theoretical and practical guidelines [22], [23]. A double layer design using setting (urban/rural) as the first layer and different audiences (adolescents, parents and school staff) as the second layer, allowed for comparison and/or verification of results between these different layers [23]. The number of focus groups was defined prior to the start of the survey [23] and considered sufficient as data saturation was reached. We conducted 20 focus groups, of which 12 were with adolescents separated by age group (6 for grade 8–9; 6 for grade 10–11) to produce homogenous groups, since ability and level of comprehension differs substantially with age [22]. In addition, 4 focus groups with parents and 4 with school staff were conducted. Participants received healthy refreshments as an incentive to participate and completed a socio-demographic questionnaire; a verbal record was taken in case of illiteracy. Audio-recorded focus groups, lasting 32 minutes on average, were conducted in Spanish and led by a trained interviewer (AO). A silent observer (RV) was present to take notes on non-verbal individual behavior and group interactions [22], [23]. Using the theoretical framework, a semi-structured questioning route was developed, pre-tested and refined. The issues addressed were designed to solicit information about the individual, physical and social eating environment of adolescents, consistent with the models selected. Open-ended questions were followed by more specific probes to clarify and extend responses. Adolescent focus groups opened with a visual listing of healthy and unhealthy foods which was then referred to during the group discussion. After each focus group, a debriefing was held with the moderator and observer.

### Participants

Focus groups were conducted in 5 schools, 3 from Cuenca (urban) and 2 from Nabón (rural), which were selected by convenience sampling. Each of the 3 schools in Cuenca represented a distinct socio-economic level, i.e. low, middle, and high. There were only schools of low socio-economic level in Nabón. Schools were categorized into these different levels based on the type of school (public/private) and school fees. From each of these 5 schools, 20 adolescents (grade 8–11) were randomly selected. Convenience sampling was used to recruit participants to the parent and school staff focus groups. To be eligible, parents needed to have a child (aged 11–15 y) at one of the participating schools and school staff had to be employed at one of the schools.

### School setting

Schools had either contact hours in the morning (7 am–1 pm; *n* = 4) or in the afternoon (12 am–6 pm; *n* = 1) and both had one break of approximately 30 minutes. Food service was provided through a privately owned tuck shop, i.e. a small food-selling retailer, based either in school (urban) or outside school (rural). Adolescents have easy access to street foods nearby school and sometimes street food vendors enter the school premises.

### Data coding and analyses

Records were transcribed verbatim, translated into English and cross-checked by 3 researchers. We used a deductive thematic content analysis [24] which was based on both the literature and the theoretical framework of this study. This enabled us to identify themes and factors influencing dietary behavior of adolescents. The purpose of identifying these themes and factors was to build up a model, i.e. a conceptual framework explaining the dietary behavior of our participants. Using this analysis, 2 investigators independently read the transcripts and identified emergent themes. For each participant group, a codebook based on these factors was developed independently by 2 researchers. If no agreement was reached on coding, a third researcher was consulted. The codebook was further validated on different transcripts. NVivo software (QSR international – version 8.0) was used to code, manage and analyze the data. Summary reports were written for each participant group according to identified factors and themes. Moreover, focus group attributes, such as socio-economic status and school setting were cross-linked with constructs and themes for each participant group. For triangulation of the data we took into account the non-verbal behavior, group interactions and data from the parent and school staff groups. Findings from the focus groups were grouped into individual and environmental factors influencing eating behavior, which were subdivided into specific factors according to the literature and the theoretical framework used. Inclusion of factors was based on the frequency, specificity, emotion and extensiveness of the quotes related to the factor [23]. Data from all participant groups are presented for each selected factor and related quotes are shown in **Tables S1 and S2** *(online material)*. The differences in these factors among the socio-economic levels and settings are only presented where relevant.

## Results

Twelve adolescent focus groups (*N* = 80) were conducted and group size ranged from 6–8 individuals. In addition, 4 parent (*N* = 32) and 4 school staff groups (*N* = 32) with an average group size of 8 were performed **(** **Table 1** **)**.

**Table 1:** Participant characteristics.

	Total	Urban	Rural	*P*-value
**Adolescents (n = 80)**				
Gender (% male)	47.9	46.1	50.0	0.63
Age (mean (SD) yrs)	13.7 (1.2)*	13.7 (1.3)*	13.8 (1.1)*	0.67
School (% public)	62.5	31.2	68.8	<0.01
Socio-economic level based on schools				
Low (%)	67.5	35	100	<0.01
Medium (%)	17.5	35	0	<0.01
High (%)	15	30	0	<0.01
**Parents (n = 32)**				
Gender (% male)	25	11	43	0.04
Age (mean (SD) yrs)	41.2 (10.7)	38.5 (6.5)	44.6 (14.1)	0.16
No. of children (mean (SD))	2.9 (1.4)	2.7 (0.9)	3.1 (1.8)	0.39
Education				
Illiterate (%)	6.5	5.5	5.6	0.001
Primary (%)	45.2	84.6	16.6	0.001
Secondary (%)	32.3	0	55.6	0.001
University (%)	16.1	7.7	22.2	0.001
**School staff (n = 32)**				
Gender (% male)	58.1	41.2	78.5	0.04
Age (mean (SD) yrs)	36.7 (11.0)	39.6 (12.6)	33.1 (7.4)	0.09
Experience (mean (SD) yrs)	7.0 (8.7)	9.6 (11.6)	4.6 (4.0)	0.15

*Date of birth was missing for 5 adolescents.

*P*-values for urban-rural differences (two sample t-test, Chi square or Fisher Exact test).

The results are presented according to the two broad levels of individual and environmental influences, identified in the analysis. Furthermore, environmental influences are presented according to the influences at school, family, and physical and societal level.

### Individual factors influencing eating behavior

#### Awareness

Adolescents mainly discussed healthy eating by identifying stereotype foods or food groups they perceived as (un)healthy, naming many more “unhealthy” than “healthy” foods. Fruit and vegetables were perceived as healthy, while French fries, potato chips, candies and ‘junk food’ (referred to as such by participants) were most frequently mentioned as “unhealthy foods”. On the other hand they mentioned, but less frequently, that eating healthily includes a balanced diet with a low amount of fat and lots of vitamins. Adolescents reported that they were aware of the general health benefits of eating healthily. They believed that traditional and home-grown foods are ‘always’ healthy as these were hygienically prepared at home. In contrast, street or restaurant foods and food out-of-home in general were perceived as unhealthy because preparation methods were unknown.

Parent and school staff groups reported that a healthy diet includes balanced and varied dietary practices in which moderate portion sizes, having breakfast, and eating regularly at set times are important. Like adolescents, they associated eating healthily with traditional, home-grown and hygienically prepared food and not necessarily with nutritional quality. Parents expressed their concerns about food safety in school tuck shops.

#### Attitudes

Overall, adolescents reported positive attitudes towards healthy eating, with some of them associating healthy eating with a positive body image and health benefits, such as looking good and being healthy. Nevertheless, they reported liking “unhealthy food” so much that they could not resist it, even though they were aware of its poor nutritional value. Parents and school staff in the study generally had positive attitudes towards healthy eating but anticipated that adolescents would hold negative attitudes.

#### Taste

Overall, adolescents were enthusiastic when talking about the taste of sweet and fatty foods, while vegetables or salads were associated with unpleasant and negative taste experiences, particularly in the school environment. As such taste had an important impact on their preferences and consumption. This was re-iterated by parents and school staff.

#### Self-efficacy

Many adolescents felt they would not succeed in eating healthily and associated this inability with lack of self-control and the abundance of tasty, yet “unhealthy food” at school and/or at home. Only a few adolescents indicated that they are or would be capable of eating healthily.

School staff groups acknowledged their responsibility in educating adolescents about healthy eating, but also stressed the importance of parental responsibility. Surprisingly, parents did not recognize their responsibility for their children's dietary behavior, but placed it with school, the environment or their children themselves.

#### Financial autonomy

Adolescents reported having financial autonomy to choose food, generally originating from pocket money received from parents/grandparents or money earned by them. This pocket money was mainly used to purchase foods of poor nutritional quality at school. Even though no differences were noted among adolescents from different socio-economic groups, parents from low socio-economic groups reported that their children did not receive any/much money and mostly took food from home to eat at school.

#### Habit strength

Most adolescent groups noted that their food consumption was influenced by habit, which they reported has become less healthy since moving to secondary school. They identified the increased availability of “unhealthy food” and (financial) autonomy as main influences on their habits. A strong habitual pattern was reported with regard to eating out at weekends.

Parents and school staff groups also saw habit strength as a key influence. They expressed concern about the changes adolescents face, such as increased (financial) autonomy and less parental control, and the transition from primary to secondary school accentuated the changes that have occurred in the socio-cultural environment over recent years.

#### Subjective norm

Views on the pervasiveness of subjective norms on healthy eating varied among adolescent groups. Most adolescents reported being afraid of what others might think if they ate healthily, such as embarrassment, being called “freaky”, “weird” or “not willing to spend money” or the possibility of being mocked by their peers. Positive perceptions were reported less often and generally these adolescents felt confident and did not care what their peers or other people thought.

Parents and school staff groups also emphasized the fear of embarrassment held by adolescents regarding eating healthily, indicating some strong social norms were operating in the peer environment.

#### Perceived barriers

Adolescents from low socio-economic schools described the cost of healthy food as a barrier to eating healthily, which was also stressed by parents. Furthermore, rural adolescents reported that availability was a barrier to eating healthily. These 2 key factors were distinct for urban adolescents who reported (as did parents) that food is readily available and cost was not an issue. Some adolescents reported lack of time as a barrier for eating breakfast at home; this view was shared by parents. All school staff and parent groups described the impact of the changing society and environment on lifestyles. Significant barriers to eating healthily at home were: having less time to prepare (healthy) meals, challenges of organizing their schedules around family meals, and choosing convenient ready-to-eat dishes which are preferred over “healthy foods”.

### Environmental influences on eating behavior

#### Family environment: parental rules, role modeling and availability

Three key factors - parental rules, availability and role modeling – were identified. Some parents reported they try to be a good role model for their children and include rules about healthy eating. Nevertheless, they confirmed that they inconsistently enforced rules about healthy eating and do not always set a good example for their children. They acknowledged that it is difficult to expect their children to eat healthily if they do not do so themselves. Parent groups reported that these inconsistencies arose from the fact that preparation and consumption of healthy food at home is very often a negotiation process with adolescents. Due to this constant struggle to encourage their children to eat healthily, parents reported often giving in and adapting meals to children's wishes. These inconsistencies were reflected in adolescents' responses who stated that they tend to disobey rules on healthy eating, particularly away from home. Nevertheless, adolescents indicated that the availability of healthy food at home had an influence on their eating pattern, because they eat what is served and available at home. Rural parents were most likely to evoke their dependency on their own food production to ensure that healthy food is available at home, whereas for urban participants this was more related to availability in shops.

#### School environment: school rules and availability

At school level, rules and availability were the 2 most important factors. Urban adolescents reported food restrictions at school, e.g. soft drinks and French fries. However, some adolescents did not feel constrained by these school rules and purchased their preferred food outside school. This was different for rural adolescents, where no restrictions on food were in place, as the tuck shop was external to the school. School staff confirmed adolescents' views on food restrictions at school and stated that these were guided by food hygiene and safety practices, rather than by nutritional quality. Food availability at school was viewed by adolescents as a key factor influencing their consumption, i.e. they eat what is available. Parent and school staff groups confirmed the abundance of ‘junk food’ and poor availability of fresh fruit at school. However, they explained that food available in the tuck shop is a reflection of adolescents' preference for processed food. Even when fresh fruit was available at the school tuck shop, it was not sold to adolescents as it was often seen as unpalatable to them. However, all participant groups believed that if fresh fruit looked appealing, was ready-to-eat and sold at an acceptable price then adolescents would be more willing to buy it. These tuck shops typically sell confectionery food, such as sweets, crisps, ice cream and soft drinks. In addition to these foods, some of them offered warm snacks or meals during the break such as ‘salchipapas’ (French fries with sausage), fried ‘empanada’ (deep-fried pastry snack) or rice with chicken/meat.

#### Environment outside home and school: socio-cultural changes and availability

Parents frequently stated that ‘junk food’ is available everywhere, not only at school, but also outside school. In addition, parents from higher socio-economic groups emphasized that media has a large impact on their children's eating habits, as food advertisements are specifically targeted towards children. Parents and school staff believed that the availability of sweets and processed foods had increased since they were young. Both evoked the impact of the changing socio-cultural environment on traditional diets, food availability and family meal patterns. All these factors have led to increased portion sizes and a variety of palatable foods with poor nutritional quality.

### Conceptual framework

**Figure 1:**
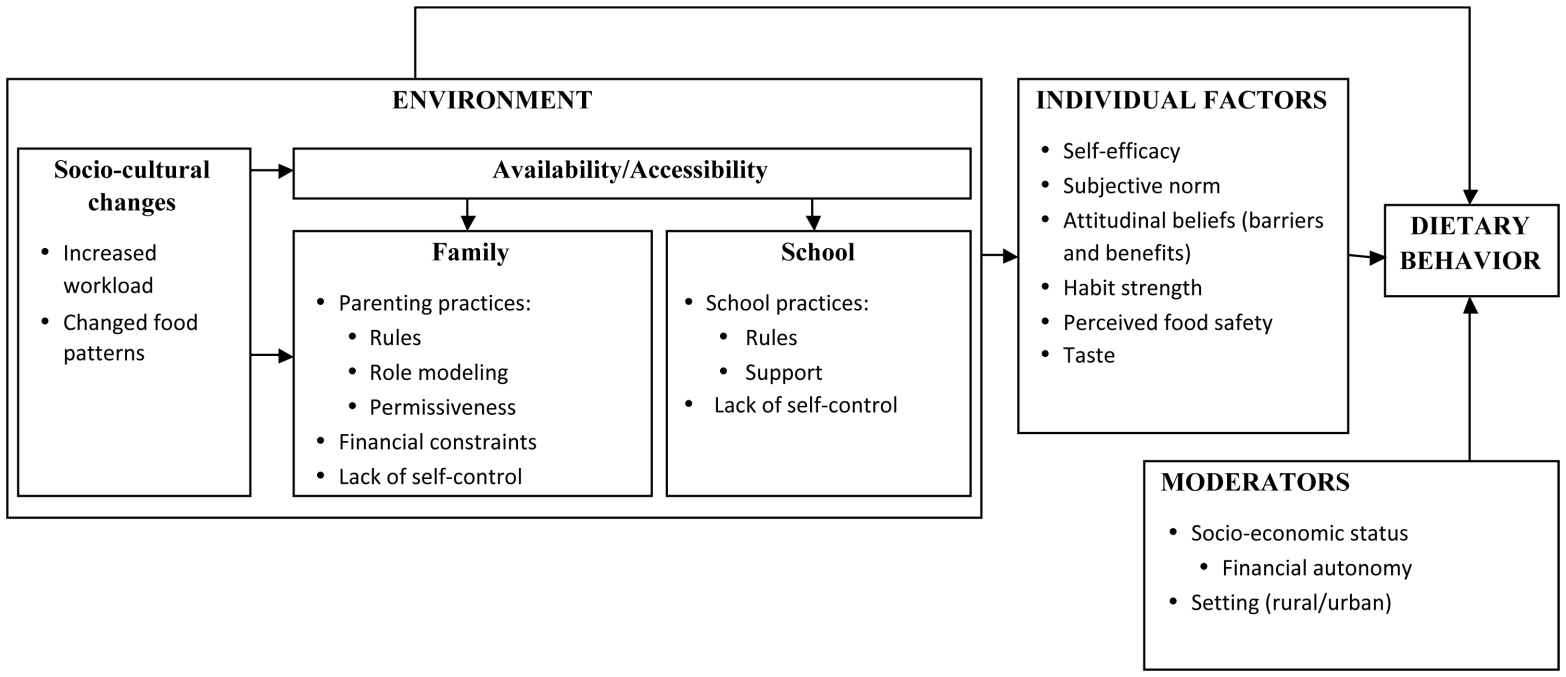
Conceptual framework for eating behavior in Ecuadorian adolescents.

Based on our findings a composite conceptual framework was proposed, in which adolescent eating behavior is conceptualized as a function of the identified individual and environmental influences (**Figure 1**). The framework emphasizes the interaction of factors within and across these levels of influence. All of these factors may directly or indirectly influence adolescents' dietary behavior. In addition to the more traditional influencing factors, the following culture-specific key factors were identified for our population: perceived food safety, lack of self-control, financial autonomy, habit strength and changes in socio-cultural environment. Furthermore, as acknowledged previously [13], our findings indicated that the influence of these factors on behavior may differ according to socio-economic status and setting. This multilevel, interactive framework is useful for understanding and explaining the factors influencing dietary behavior in Ecuadorian adolescents.

## Discussion

Eating behavior is influenced by inter-related factors reflecting ones' personal, social and cultural experiences and environment [25], [26]. In addition, the reasons for choosing particular foods are closely associated with concerns over identity, image and social belonging [26], which is ubiquitous in adolescence. Several culture-specific key factors - perceived food safety, lack of self-control (attribution error), financial autonomy, habit strength and changes in socio-cultural environment - emerged from focus groups endorsing the importance of the development of a conceptual framework in this population for future interventions.

First, participants often associated eating healthily with food safety issues and home-grown or -prepared food rather than with nutritional quality of their diet as a whole, which had an impact on which foods were prohibited at school and on adolescents' food choices. The importance of food safety in defining eating healthily has been noted in previous research as an important factor for LMICs [27].

Whilst school staff recognized their role in shaping adolescents' dietary behavior, they minimized their responsibility. They saw parents as gatekeepers of adolescents' poor eating habits, suggesting that parents need to act as positive role models. However, parents evoked their work commitments, the changing socio-cultural environment, schools and their children's food preferences as key influences on food choice. This concept of attribution error, i.e. blaming situational factors when justifying one's behavior, has been demonstrated previously [28].

Increasing financial autonomy, which coincides with the transition from primary to secondary school, played a large part in adolescents' food choices. This confirms previous findings in Vietnamese adolescents, where pocket money increased eating out frequency [29]. Starting secondary school is a critical period of increasing independence as the extent of parental support for eating healthily decreases and the desire to fit in with peer norms increases [18]. This process, in conjunction with easy access and constant exposure to tasty and unhealthy food in schools, explains the adolescents' indication of their deteriorating eating habits. This deterioration is accentuated further by the increasing difficulty adolescents have to eat healthily within the rapidly changing socio-cultural environment, which has impacted on family life and food availability, mirroring the ongoing nutrition transition in Ecuador [30] and other LMICs. Ready-to-eat meals in large portion sizes are now the norm, due to busy family work schedules. A similar pattern has occurred in other countries where women's roles have changed, leading to a loss of cooking skills and an increasing reliance on convenience foods [31]. A daunting prospect, as eating out and relying on convenience foods has been associated with poor dietary intake in LMICs [32].

In line with previous findings from HICs, taste [33], availability and accessibility [27], [34], self-efficacy, financial constraints, time and convenience [25], [34] emerged as important features in adolescents' food choices. In addition, strong subjective peer norms were present - choosing to eat healthily was often associated with an untrendy image leading to teasing from others and marginalization - supporting the preferences for unhealthy foods of adolescents. Similarly, Stead *et al.* (2011) found that *“it’s emotionally and socially risky to be seen to be interested in healthy eating”* for adolescents in school and peer contexts [26]. To conclude, rules at home and at school were inconsistent, so adolescents were likely to receive contradictory messages that they regarded as marginal and they developed strategies for buying their preferred food elsewhere. This might be an indirect indication that parental influence is less important in this group than peer influence. Similar associations between mixed messages and adolescent eating preferences have been found in previous research [35]. Nevertheless, the impact of parents might differ across behaviors (e.g. fruit and vegetable consumption versus sugary drink intake) [36].

Few socio-demographic differences emerged. Availability and financial constraints clearly differed between the rural and urban area and the socio-economic groups, supporting findings from previous focus groups in LMICs [27]. These differences might explain why participants from rural and low socio-economic schools reported lower availability of healthy food at home and could not afford to buy “healthy foods”. Previously, the importance of socio-demographic factors as moderating factors or effect modifiers of behavior has been established [37], [38]. This means that influencing factors may have differential effects on behavior with respect to socio-economic status [13], [39], [40] and setting [41], which supports the inclusion of these as moderating factors in the conceptual framework.

Adolescent participants might have experienced difficulties in sharing their views within the focus groups due to social desirability and peer pressure. Yet, we do not believe this influenced our results to a great extent, as the moderator tried to establish a friendly and comfortable environment encouraging active participation and secondly, and more importantly, findings did not differ across adolescent groups. We aimed at minimizing bias by using triangulation and standardized data collection procedures. Since parents and school staff re-iterated the findings of the adolescent focus groups we can assume these findings are valid. Furthermore, despite the accumulating evidence of unhealthy dietary practices, dietary behavior remains poorly understood in young people in LMICs [37]. Few attempts have been made to use theory to guide the development and evaluation of interventions [12]. Additionally, testing the validity of these theories, i.e. their appropriateness to specific cultures and local contexts, is rarely undertaken [42]. This study adds to the current evidence-base, by identifying key factors influencing Ecuadorian adolescents' eating behavior and developing a composite conceptual framework. The factors identified within this framework should be investigated using culturally appropriate scales with good psychometric properties. Doing so would allow this framework to be tested by evaluating the inter-relationships and association of these factors with dietary behaviors. Additionally, it facilitates tailoring of intervention strategies towards these factors, and could be used to identify pathways of behavior change when evaluating interventions [42].

Our conceptual framework indicates that future interventions should not only consider individual, peer and family influences when aiming to change adolescent eating habits, but should target the physical school and social environment as well, which is consistent with findings from other studies [43]. A particular focus on school policies including regulation on food sold at the tuck shop based on its nutritional value and control of food practices is needed. Such strategies need to be tailored to the specific settings and socio-economic conditions, even though this might be challenging [12]. Specifically, the intervention should take into account the issue of attribution error amongst parents and school staff. Despite the possible relative importance of parents, they still play an important role in the daily life and dietary behavior of adolescents and should be included when designing interventions [38], particularly in LMICs [12]. On a positive note, all participant groups requested practical advice on how to eat healthily and develop skills.

## Conclusion

Focus groups provided a clear insight into the factors that influence adolescents' dietary behavior. Adolescents, parents and school staff identified financial autonomy, food safety, self-efficacy, habit strength and socio-cultural changes as key cultural factors in adolescent's food choices. As a consequence, a conceptual framework for adolescents' eating behaviors emerged, which highlights points of leverage for developing future interventions. Interactions between the identified factors in the conceptual framework and eating behaviors should be studied using structural equation or mediation analysis.

## Supporting Information

Table S1 **Quotes on individual factors influencing eating behavior in adolescents, parents and school staff.** A: adolescents; P: parents; S: school staff; …: short silence; […]: overlapping speech; __: emphasis; £ £: smiley voice; §§: laughing; / /: irony; (()): transcribers' comments.
(DOCX)

Table S2 **Quotes on environmental factors influencing eating behavior in adolescents, parents and school staff.** A: adolescents; P: parents; S: school staff; …: short silence; […]: overlapping speech; __: emphasis; £ £: smiley voice; §§: laughing; / /: irony; (()): transcribers' comments.
(DOCX)
