# Supplementary material for: A Conceptual Framework for Healthy Eating Behavior in Ecuadorian Adolescents: A Qualitative Study
Source: PLoS One. 2014 Jan 29;9(1):e87183. doi: 10.1371/journal.pone.0087183 (PMC3906122; doi:10.1371/journal.pone.0087183)
Supplement: Table S1 — Quotes on individual factors influencing eating behavior in adolescents, parents and school staff. A: adolescents; P: parents; S: school staff; …: short silence; […]: overlapping speech; __: emphasis; £ £: smiley voice; §§: laughing; / /: irony; (()): transcribers' comments. (DOCX) [file pone.0087183.s001.docx]

Table S1 Quotes on individual factors influencing eating behavior in adolescents, parents and school staff

| **Factor** | **Quotes** |
| --- | --- |
| **Awareness** | A1: “To have a better life and be able to live longer.” |
|  | A2: “£That we are strong, sexy£.” |
|  | A3: “I think that it is unhealthy because the preparation is not hygienic, the food could be contaminated.” |
|  | P1: “To have at least 5 meals per day…for their age. To have a good breakfast and always include a dairy product, and a cereal, cheese, eggs. And to vary the food and don’t have all the time the same. Something for snack in the morning, a healthy snack…for lunch…always a healthy lunch that includes vegetables, salad, protein, meat, fruits…and in the afternoon fruits and…like…a good dinner…to don’t skip meals, as a habit to eat every day in the same way, at the same time.” |
|  | P2: “… but I am also worried about how things are done here. Because potatoes are fried in the same oil, they reuse the oil for a week. I have discussed this with the teachers, because one person has to go and check every day that the oil has been changed. I am really worried.” |
|  | S1: “We have assessed reintroducing our traditional foods like barley, quinoa, potatoes, machica … it would be important for the students to eat that.” |
|  | S2: “ ‘Gatorade [energy drink]’ is forbidden because we received information that some substances affect the kidneys. Thus the director at that time met with the parents and asked to stop giving Gatorade to the kids, also and other things… because this information has popped up.” |
| **Attitudes** | A1: “Once in a while it is also good to eat a bit healthy …because if you eat only meat … you can get sick.” |
|  | A2: “It is not healthy but tasty §§.” |
|  | P1: “With the vegetables, we have to explain to them, over and over… they never say ‘oh this is tasty’ . Never. Like tasty broccoli or spinach... nooooo.” |
|  | P2: “How can we be completely sure that the children eat what we give them? That is not real. I used to give him fruit for school, he used to sell it and with that money bought foods at the grocery.” |
|  | S1: “Well, the youngsters … drink a glass of soft drink, … they prefer that.” |
|  | S2: “The adolescents think that they don’t need to eat breakfast.” |
| **Taste** | A1: “I don’t … I prefer … the salads at home they taste different, here I don’t like them, I prefer the tasty ones, here they taste different.” |
|  | A2: “I don’t like egg plant … wuuuak ((he makes a noisy gesture referring to nasty))” |
|  | P1: “£ They always drink soft drinks, but I also like it huffff £” |
|  | S1: “Yes, they don’t like it because it’s healthy food.” |
| **Self-efficacy** | A1: “I wouldn’t do it because it is difficult as there are a lot of places and stores in which I can buy unhealthy food.” |
|  | A2: “I mean, I eat healthily because I … I like it, I don’t like to be fat, I don’t know… for that reason I control myself… I don’t drink soft drinks… nothing like that… I don’t like mayonnaise.” |
|  | P1: “As I mentioned they are influenced by others…” |
|  | S1: “It would be important to motivate healthy eating at home. Because it depends on themselves, in my case my daughters … it depends on them that they come to school having breakfast. Doesn’t it. They have had this habit since they were little, thus I know they will keep on doing it, and for the next generations. It really depends on how we, the parents, have taught them.” |
|  | S2: “Well, I think we can provide some things, but adequate nutrition is most feasible at home.” |
| **Financial autonomy** | A1: “When we are paid, when we work … Friday I will have a party and get well paid… I entertain parties for kids, and I get money that I use to buy all that I want.” |
|  | A2: “When I get money, I go and buy French fries.” |
|  | P1: “My children do not eat in the school break, because we give them 50 or 60 cents per day, but they save that … especially the girl, but the little boy spends all his money buying hot dogs, pizza at the bar. But the girls do it once in a while.” |
|  | P2: “I give them money for food on the break.” |
| **Habit strength** | A1: “When we go to feed the cattle, we chew gum, lollipops.” |
|  | A2: “…when we were little our parents used to control what we ate or not, we used to eat mostly at home. But now we go out with friends and nobody checks if we eat fat.” |
|  | P1: “Some kids are used to drinking soft drinks, and they complain. It is difficult for a kid to drink juice when he has been used to drink soft drinks…that is difficult to handle. It is a lifestyle, they are used to it.” |
|  | P2: “… we don’t eat together anymore. We used to eat together at the same time. It has changed when they started secondary school, the academies… I think there will be negative results.” |
|  | S1: “At school the kids decide, more or less, what to eat…[…..] This does not happen at primary school, because there, they receive their meal…that is …controlled. But at secondary school it’s a little bit more difficult, because the adolescents don’t receive a meal. Therefore, what they should eat is a bit more fruit, but they don’t like it, they don’t eat it.” |
| **Subjective norm** | A1: “They would make fun of me, they would say that we are tight… that we do not want to spend money.” |
|  | A2: “Because, I get embarrassed of eating rice in front of my classmates.” |
|  | A3: “I don’t think they would say anything, it is my life and I choose what to eat, can’t I?” |
|  | P1: “She brings …carrots, milk or fruit … she is seen as a freak.” |
|  | P2: “I tried to give them ((fruit)) when they were little, for example apples … but they say they get embarrassed… I never did it again.” |
|  | S1: “When she is seen thin, and we haven’t seen her in this condition from the beginning…or when she’s getting fat … We ask them … what happened to you? Aren’t you doing enough exercise? Are you eating a lot? Or something like that… We intervene making comments like these, that is our relation… we have to take care of our students...” |
|  | S2: “… She said ‘no’, that she felt embarrassed of eating that ((‘junk food’)), then it was very difficult, because it is quite popular and cool to eat ‘junk food’.” |
| **Perceived barriers** | A1: “Because we don’t buy the food we don’t like…or because sometimes it is not at the stores.” |
|  | A2: “Because there is no money to buy it.” |
|  | A3: “Because sometimes it [‘junk food’] is the easiest and fast to cook.” |
|  | P1: “They don’t think, they just eat what is available and convenient. We don’t know if that is good or bad.” |
|  | P2: “The convenience of the new things ((foods)).” |
|  | S1: “Because before … I am not sure but, it could be that parents used to care more about eating than now…and now it is easier to get ready-to-eat food, which can be bought and eaten. Hamburgers and all these things make kids fat…and don’t let them grow. They eat less vegetables…etc. I think it’s the convenience of frozen foods, because before mothers used to be at home cooking and sometimes stay at the table for half an hour to wait until the kid eats everything. Therefore, habits have changed…for time limitation and convenience. These are the reasons why things have changed a lot.” |

A: adolescents; P: parents; S: school staff; …: short silence; […]: overlapping speech; : emphasis; ££: smiley voice; §§: laughing; / /: irony; (()): transcribers’ comments
