# Supplementary material for: A Conceptual Framework for Healthy Eating Behavior in Ecuadorian Adolescents: A Qualitative Study
Source: PLoS One. 2014 Jan 29;9(1):e87183. doi: 10.1371/journal.pone.0087183 (PMC3906122; doi:10.1371/journal.pone.0087183)
Supplement: Table S2 — Quotes on environmental factors influencing eating behavior in adolescents, parents and school staff. A: adolescents; P: parents; S: school staff; …: short silence; […]: overlapping speech; __: emphasis; £ £: smiley voice; §§: laughing; / /: irony; (()): transcribers' comments. (DOCX) [file pone.0087183.s002.docx]

Table S2 Quotes on environmental factors influencing eating behavior in adolescents, parents and school staff

| **Factor** | **Quotes** |
| --- | --- |
| ***Family environment*** | |
| **Parental rules** | A1: “At lunch, they tell me that I have to eat at the school tuck shop, and a milk at the break, something like that, healthy, but £I don’t do it £.” |
|  | A2: “My mother advises me, but she doesn’t oblige me.” |
|  | P1: “… and we are used to letting them eat what they want on the weekends.” |
|  | P2: “When we are at home, they ((children)) decide on what to eat.” |
| **Availability** | A1: “I eat healthy at home because my parents buy or cultivate this kind of food.” |
|  | A2: “When my mother goes to the market I eat lots of fruit.” |
|  | P1 “Well, we give them breakfast, and at school... sometimes they get school lunch… otherwise they buy at the grocery shop here… French fries, these things.” |
|  | P2: “Well, some of us, we do not have plants ((growing)), but if we have… for example apples, we eat them all the time. We carry them in our pockets, it depends …well we cannot say we have them and the others don’t… but we haven’t dedicated ourselves to cultivate…for example, I have, but this season is ending… but from February until May, three months, they eat when they want. After the season we have to buy, that is how it is.” |
| **Modeling** | A1: “On the other hand my grandparents teach me, but they eat more dessert and things like that.” |
|  | A2: “If I see other people eating unhealthy I want to do it too.” |
|  | P1: “If the mother cannot stop drinking soft drinks, the kids won’t neither.” |
|  | P2: “… I think that the environment is affecting her a lot, I don’t know why she is obsessed controlling her weight, and she is thin. That is something I am very worried about, I think she sees the models on TV, she sees her friends. But for example, I give them rice in the afternoon but at night I always gave them chocolate milk, but now … she doesn’t want the chocolate, just pure milk. She is the only one that asks for pure milk, but I noticed that it’s to avoid the sugar and the cocoa. So I see that should come from her environment… The boy on the other hand, wants treats and to eat what all the friends eat, like plantains, Goudies ((potato chips)), Cachitos ((potato chips)).. thus he is not going to ask for an apple … even if I send him fruit from home, or because I said that fruits are healthy.” |
| ***School environment*** | |
| **School rules** | A1: “Bubble gum, bubble gum is not sold anymore.” |
|  | A2: “… almost everything can be found at the tuck shop.” |
|  | P1: “From last year French fries and sausages… because they use a lot of saturated fats” |
|  | P2: “That is good, they used to use the same oil. They kept it in bottles and reused it and with that they used to make the sauce.” |
|  | S1: “I am sure that school is the only high school in Cuenca that does not sell saturated fatty foods, [yes]. In that sense we are novel, we have avoided color additives consumption in drinks, there is no Manzana drink ((Ecuadorian soft drink)), only Sprite is being sold and juices… fruit juices such as blackberry juice, orange juice… The children have got used to drink juices or yogurt. We also broke the contract with Coca Cola… because it is not drunk anymore.“ |
| **Availability** | A1: “At school we eat unhealthily because we can only buy this type of food and because we like it.” |
|  | P1: “They tend to buy treats, thus it wouldn’t be worth the bar selling fruit or healthy foods… it’s not worth. They like potato chips, cassava, plantains… all fried.” |
|  | S1: “What is sold on the school grocery are sandwiches, tacos with avocado and tomato, French fries and sausage, hamburgers, French fries, hot dogs, Cuban sandwiches, and always potato chips, cookies, chips.” |
| ***Outside home and school*** | |
| **Availability** | A1: “Sometimes when we are out, and we are at the grocery …and there are chocolates.” |
|  | P1: “Unfortunately, not all of us do that ((serve balanced meals)). Even if we want and do our best, we can’t. Specially due to all kinds of ‘junk food’ around these days” |
|  | P2: “Other problem is that when we were little, there was not too many treats compared with now. There were, but not as much as today. And I see that the children eat it a lot.” |
|  | S1: “Ahhh yes, sure here in front of the school… hamburgers are sold for one dollar [with fries and everything]… § well all that has fats §.” |
| **Socio-cultural changes** | P1: “I think the publicity is aggressive and directed towards them…” |
|  | P2: “I think, bad feeding comes from the media, the TV etc. |
|  | P3: ““It is a lifestyle, they are used to it. It’s not the lifestyle …as we were used to, we used to sit to eat at 1pm and have time to calm down” |
|  | S1:“Now with modernization, the microwave … now everybody comes, put in the microwave and … runs. The cooking habit has been lost” |
|  | S2: “Unfortunately, we are driven by a consumer society where lots of fast products are offered and sold…” |

A: adolescents; P: parents; S: school staff; …: short silence; […]: overlapping speech ; : emphasis; £ £: smiley voice; §§: laughing; / /: irony; (()): transcribers’ comments
